# Markers of fibroblast-rich tumor stroma and perivascular cells in serous ovarian cancer: Inter- and intra-patient heterogeneity and impact on survival

**DOI:** 10.18632/oncotarget.7613

**Published:** 2016-02-23

**Authors:** Sara Corvigno, G. Bea A. Wisman, Artur Mezheyeuski, Ate G.J. van der Zee, Hans W. Nijman, Elisabeth Åvall-Lundqvist, Arne Östman, Hanna Dahlstrand

**Affiliations:** ^1^ Department of Oncology-Pathology, Karolinska Institutet, Stockholm, Sweden; ^2^ Unit for Breast, Gynecologic Cancer and Sarcoma, Department of Oncology, Karolinska University Hospital, Stockholm, Sweden; ^3^ Department of Gynecologic Oncology, University of Groningen, University Medical Center Groningen, Groningen, The Netherlands; ^4^ Department of Oncology and Department of Clinical and Experimental Medicine, Linköping University, Linköping, Sweden

**Keywords:** serous ovarian cancer, tumor microenvironment, cancer associated fibroblasts, pericytes, prognosis

## Abstract

Inter- and intra-patient variations in tumor microenvironment of serous ovarian cancer are largely unexplored. We aimed to explore potential co-regulation of tumor stroma characteristics, analyze their concordance in primary and metastatic lesions, and study their impact on survival. A tissue microarray (TMA) with 186 tumors and 91 matched metastases was subjected to immunohistochemistry double staining with endothelial cell marker CD34 and fibroblast and pericyte markers α-SMA, PDGFβR and desmin. Images were digitally analyzed to yield “metrics” related to vasculature and stroma features.

Intra-case analyses showed that PDGFβR in perivascular cells and fibroblasts were strongly correlated. Similar findings were observed concerning α-SMA. Most stroma characteristics showed large variations in intra-case comparisons of primary tumors and metastasis. Large PDGFβR-positive stroma fraction and high PDGFβFR positive perivascular intensity were both significantly associated with shorter survival in uni- and multi-variate analyses (HR 1.7, 95% CI 1.1-2.5; HR 1.7, 95% CI 1.1-2.8).

In conclusion, we found PDGFβR- and α-SMA-expression to be largely independent of each other but concordantly activated in perivascular cells and in fibroblasts within the primary tumor. Stromal characteristics differed between primary tumors and metastases. PDGFβR in perivascular cells and in fibroblasts may be novel prognostic markers in serous ovarian cancer.

## INTRODUCTION

Ovarian cancer is the deadliest gynecological malignancy worldwide, with an overall poor survival. At present, histology, stage, and residual disease after primary surgery are the most important factors used to evaluate the probability of survival, but the need of new and improved prognostic markers remains [1].

Ovarian tumors are characterized by heterogeneous histology, with the serous subtype being the most common [2]. Serous ovarian tumors are often composed of poorly differentiated tumor cells, named high-grade according to the two tier system [3, 4]: the high-grade subtype evolves rapidly, harbors p53 mutations [5], is characterized by genetic and epigenetic alterations of homologous recombinant pathway genes [6] and is most often diagnosed at an advanced stage [7]. Most ovarian tumors spread throughout the peritoneum [8] and metastases in the omentum typically occur early in tumor progression.

Ovarian stroma plays an essential role in the normal functioning of the organ, supporting follicle growth and development [9, 10]. Recent data indicate that the stroma may be important for tumorigenesis [11] and invasiveness in ovarian cancer due to the paracrine interaction between cancer-associated fibroblasts (CAFs) and ovarian cancer cells [12-15].

CAFs are a heterogeneous population of fibroblast-like cells that affect migration and invasiveness of tumor cells in different types of tumors [16, 17]. Differential expression of markers such as alpha smooth muscle actin (α-SMA), platelet derived growth factor beta receptor (PDGFβR), podoplanin and fibroblast activation protein (FAP) [18, 19] may account for different features and functions.

Pericytes are stroma cells that surround small vessels; they are embedded in the basement membrane in tight contact with endothelial cells [20, 21]. In normal tissue, they express markers like PDGFβR, neural/glial antigen 2 (NG2) and desmin. Tumor vessels display a phenotype with loosely attached pericytes that commonly express a different pattern of markers, including α-SMA [17]. The intimate contact with endothelial cells allows a tight paracrine interaction between the two cell types, an interaction that governs vessel function and vessel maturation. Recent studies have shown the importance of pericytes for endothelial cell survival, vessel wall stabilization, and blood flow normalization [22, 23]. Tissue-based analyses of pericytes most commonly rely on the use of a single marker such as PDGFαR, PDGFβR, α-SMA, desmin, NG2, or RGS5 [24, 25]. Current researches show that these markers can be expressed in a non-overlapping manner, an expression patterns that may reflect subtypes of pericytes characterized by different functions [26].

In this retrospective study, we applied an innovative multiparametric technique with digitalized image analysis to determine in a quantitative manner 13 different tumor stroma characteristics in a cohort of serous ovarian cancer patients. The aims were to explore potential co-regulation of these characteristics, to analyze their concordance in primary and metastatic lesions and to define their correlation with survival.

## RESULTS

### Patients

The median age for the 186 participants was 60 years (range 22 to 84 years). All patients had been diagnosed with serous ovarian cancer, 87% had FIGO stage III-IV and in 53 % the histologic grade was poorly differentiated (Table 1). All patients underwent primary debulking surgery and were followed-up until July 2006 when 59 patients were still alive (median follow-up of 51 months). Median follow-up for the whole cohort was 28 (0.03-163) months.

**Table 1:** Characteristics of serous ovarian cancer patients

Characteristic	Patients N=186
Median age, years (range)	60 (22-84)
FIGO stage I II III IV Unknown	10 (5.4%) 13 (7.0%) 130 (69.6%) 32 (17.2%) 1 (0.5%)
Histologic type Serous	186 (100%)
Histologic grade Well differentiated (grade 1) Moderately differentiated (grade 2) Poor differentiated (grade 3) Unknown	21 (11.3%) 51 (27.4 %) 98 (52.7%) 16 (8.6%)
Residual tumor after primary surgery No residual tumor Residual tumor Unknown	27 (14.5%) 102 (58.4%) 57 (30.6%)
Median follow-up time, months (range)	28 mo (0.03-162.5)
Survival Alive Dead	59 (31.7%) 127 (68.3%)

### Intra-tumor correlations of stroma markers in primary ovarian tumors

Features such as vessel density, pericyte status and CAF-marker expression have been shown to display clinically relevant variation in previous single marker studies of ovarian cancer, and other tumor types, but it is unknown if these features always change together or can change independently. To address this question we collected data on 13 different “metrics” related to the vasculature, perivascular cells, and CAFs in ovarian cancer and analyzed the intra-tumoral associations between these markers in the primary site of ovarian cancer.

**Figure 1:**
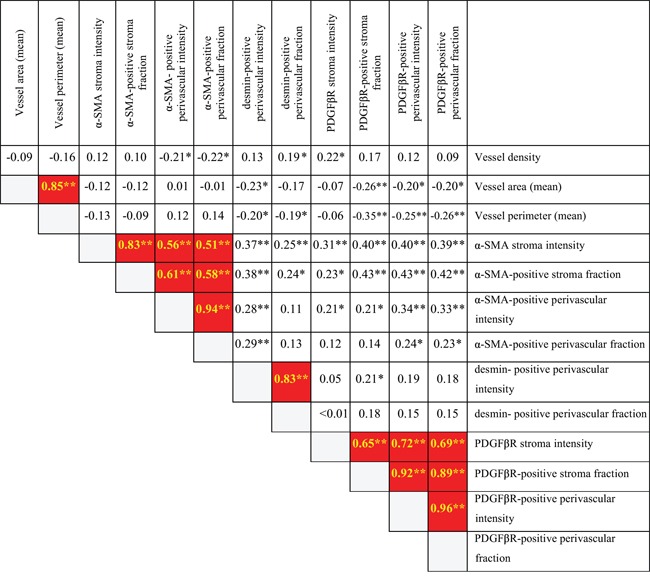
Internal correlation among stroma metrics in primary ovarian site Spearman two-tailed test shows α-SMA and PDGFβR stroma metrics correlation with the respective perivascular metrics. 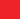 =Associations in red marked squares are with *p*-value of less than 0.01 together with a correlation coefficient higher than 0.5 (possible biologically meaningful associations). * = p<0.05. **=p<0.01.

Notably, vessel density, vessel lumen area and vessel lumen perimeter were independent of all perivascular related metrics, indicating that perivascular status is controlled by other factors than those determining vessel abundance and size (Figure 1). Furthermore, the perivascular α-SMA, desmin and PDGFβR status were also largely independent suggesting that the expression of these markers can increase or decrease independently possibly in distinct cell subsets (Figure 1). Independent expression of PDGFR and α-SMA markers was also observed in the fibroblast stroma compartment.

In contrast, α-SMA positive perivascular cell metrics and PDGFβR positive perivascular metrics correlated strongly with α-SMA positive stroma metrics and PDGFβR positive stroma metrics, respectively (α-SMA stroma fraction correlated with α-SMA positive perivascular cells intensity corr. coeff. 0.61, p<0.001, Figure 1; PDGFβR positive perivascular intensity correlated with PDGFβR positive stroma fraction, corr. coeff. 0.92 p<0.001, Figure 1).

These results suggest that the marker status of stroma fibroblasts and perivascular cells is under common control, and that these two stroma cell types may have been be derived from the same cell of origin.

### Stroma markers in ovarian primary tumor versus its metastasis

Emerging evidence indicates the presence of intra-individual differences in mutation status and chromosome aberrations between primary tumors and metastatic lesions [27]. The extent to which similar differences occur regarding characteristics of the tumor microenvironment has not been determined. Stroma features were therefore analyzed in matched primary ovarian tumors and metastatic tissue in 91 patients with serous ovarian cancer.

As shown in Table 2 the different stroma metrics showed large variation with regard to their status in primary tumors and metastatic lesions. Using a cut-off of p<0.01, three out of four of the PDGFβR-related metrics showed a correlation between primary tumors and metastatic lesions. Notably, vessel density status in primary tumor was not significantly correlated with vessel density status in the metastatic lesions.

**Table 2:** Correlation analysis between stroma tissue metrics among cases of pairwise primary tumor and metastatic lesions

Stroma tissue metrics	Corr. coeff. ovary vs metastasis (n 91)	p-value (spearman)
Vessel density	0.208	0.063
Mean vessel lumen area	0.132	0.265
Mean vessel lumen perimeter	0.279	0.012
ASMA positive stroma intensity	0.047	0.701
ASMA positive stroma fraction	−0.031	0.806
ASMA positive perivascular intensity	0.210	0.089
ASMA positive perivascular fraction	0.201	0.103
DESMIN positive perivascular intensity	0.292	0.018
DESMIN positive perivascular fraction	0.287	0.02
PDGFβR positive stroma intensity	0.474	<0.001
PDGFβR positive stroma fraction	0.341	0.004
PDGFβR positive perivascular intensity	0.414	<0.001
PDGFβR positive perivascular fraction	0.287	0.015

The analysis thus reveals, in general, that the nature of tumor stroma at the primary site differs greatly from the metastases at the intra-individual level. Additionally, the conservation of stromal PDGFβR status implies that these features may be controlled by factors acting at both the primary and the metastatic sites.

### Impact of stroma markers on overall survival

To investigate the potential utility of the stroma characteristics as biomarkers for prognosis, the associations between the 13 stroma metrics and survival were analyzed. The survival analysis of the 138 patients with available primary ovarian tissue showed that high PDGFβR positive stroma fraction (continuous variable) is correlated with a decrease in overall survival, both in the univariate analysis (HR 1.02; 95% CI, 1.01 – 1.04, p=0.01) and in the multivariate analyses after adjusting for stage, histologic grade, age and residual disease after primary surgery (HR 1.03; 95% CI, 1.01 – 1.05, p=0.004).

**Figure 2:**
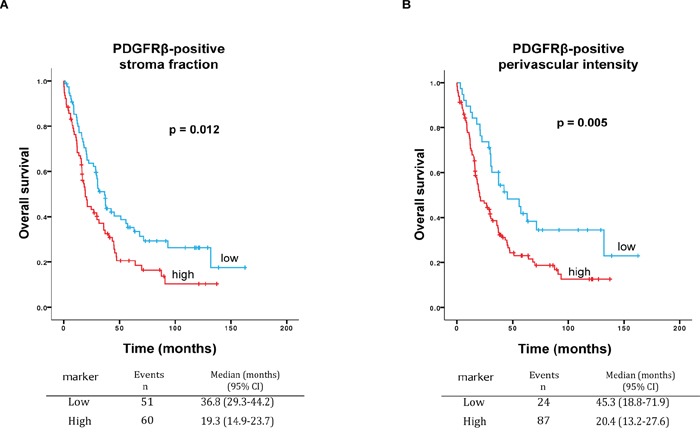
**A.** Survival curves for high and low PDGFβR positive stroma fraction. Kaplan-Meier graph shows worse overall survival for high PDGFβR positive stroma fraction as compared to low PDGFβR positive stroma fraction in serous ovarian cancer (n=186 patients) (p=0.012, Log Rank). Median survival for high PDGFβR positive stroma fraction 19.3 months versus 36.8 months for low PDGFβR positive stroma fraction. **B.** Survival curves for high and low PDGFβR positive perivascular intensity. Kaplan-Meier graph shows worse overall survival for high PDGFβR positive perivascular intensity as compared to low PDGFβR positive perivascular intensity, in 186 patients (p=0.005, Log Rank). Median survival for high PDGFβR positive perivascular intensity was 20.4 months versus 45.3 months for low PDGFβR positive perivascular intensity.

In an extended analysis, we added patients for which we lacked tumor material from the ovary but had available tissue from metastatic sites (all retrieved during primary surgery). In this extended cohort, the survival analysis was performed only on the parameters that showed concordance in the Spearman correlation test in matched primary and metastatic tissues (Table 2). In the analysis of the 186 patients with material available either from ovary (n 138) or metastatic tissues (n 48), a high PDGFβR positive stroma fraction was confirmed to be significantly correlated with lower survival rate with a median survival of 19.3 months versus 36.8 months for cases with low PDGFβR positive stroma fraction, p=0.012, Log Rank test, mean dichotomized values, Figure 2A). In concordance, Cox regression univariate and multivariate analysis showed an association between high PDGFβR stroma fraction and poor prognosis (HR 1.6; 95% CI, 1.1-2.3, p=0.01 and HR 1.7; 95% CI, 1.1-2.5, p=0.01, respectively (Table 3)). Separate sub-group analyses indicated that the prognostic significance of PDGFβR positive stroma fraction was particularly strong in the subgroup of patients that did not undergo a complete (residual tumor > 1mm) debulking surgery (HR 1.8; 95% CI 1.1-2.8, Supplementary Figure 2).

**Table 3:** Uni- and multivariate analyses of the impact of each clinical prognostic variable and PDGFβR positive stroma fraction on overall survival

Variables	Univariate analysis		Multivariate	
	HR (95% CI)	*p*-value	HR (95% CI)	*p*-value
Age at diagnosis	1.02 (0.99-1.03)	0.083	0.99 (0.97-1.01)	0.34
FIGO stage I+II III IV	1 (reference) 5.08 (2.22-11.66) 9.19 (3.75-22.54)	<0.001 <0.001	1 (reference) 2.58 (0.94-7.11) 4.11 (1.35-12.46)	0.07 0.013
Histologic grade Grade 1 Grade 2 Grade 3	1 (reference) 1.81 (0.89-3.68) 2.84 (1.46-5.53)	0.1 0.002	1 (reference) 1.11 (0.46-2.67) 1.32 (0.59-2.95)	0.8 0.5
Residual tumor after primary surgery No residual tumor Residual tumor	1 (reference) 8.57 (3.85-19.1)	0.001	1 (reference) 4.68 (1.9-11.53)	0.001
PDGFβR positive stroma fraction Low PDGFβR High PDGFβR	1 (reference) 1.61 (1.11-2.34)	0.01	1 (reference) 1.66 (1.11-2.46)	0.01

Abbreviations: HR=hazard ratio, CI=confidence interval

In the set of 186 patients, high intensity of PDGFβR perivascular staining was also found to be correlated with worse survival, with a median survival of 20.4 months versus 45.3 months for low PDGFβR positive perivascular intensity (p=0.005, Log Rank test, the lowest quartile compared to the other quartiles, Figure 2B). In concordance, Cox regression univariate and multivariate analysis showed an association between high intensity of PDGFβR perivascular staining and worse survival (HR 1.9; 95% CI, 1.2-2.9, p=0.006 and HR 1.7; 95% CI 1.1-2.8, p=0.03, respectively (Table 4)).

**Table 4:** Uni- and multivariate analyses of the impact of each clinical prognostic variable and PDGFβR perivascular intensity on overall survival

Variables	Univariate analysis		Multivariate analysis	
	HR (95% CI)	*p*-value	HR (95% CI)	*p*-value
Age at diagnosis	1.02 (0.99-1.03)	0.083	0.99 (0.97-1.01)	0.53
FIGO stage I+II III IV	1 (reference) 5.08 (2.22-11.66) 9.19 (3.75-22.54)	<0.001 <0.001	1 (reference) 2.63 (0.97-7.17) 4.29 (1.43-12.84)	0.06 0.009
Histologic grade Grade 1 Grade 2 Grade 3	1 (reference) 1.81 (0.89-3.68) 2.84 (1.46-5.53)	0.1 0.002	1 (reference) 1.01 (0.42-2.42) 1.19 (0.54-2.66)	0.98 0.66
Residual tumor after primary surgery No residual tumor Residual tumor	1 (reference) 8.57 (3.85-19.1)	<0.001	1 (reference) 4.44 (1.8-10.96)	0.001
PDGFβR positive perivascular intensity Low PDGFβR High PDGFβR	1 (reference) 1.89 (1.2-2.98)	0.006	1 (reference) 1.72 (1.07-2.75)	0.03

Abbreviations: HR=hazard ratio, CI=confidence interval

**Figure 3:**
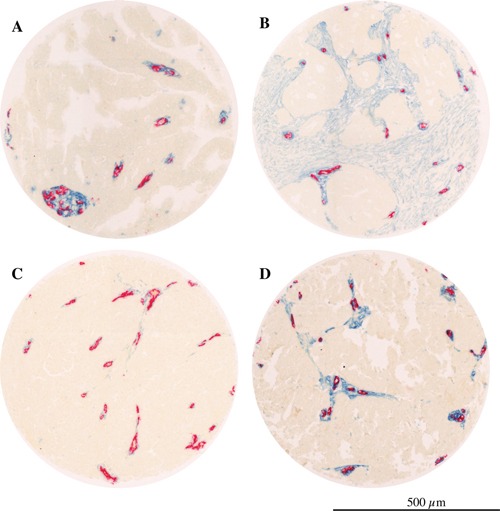
PDGFβR expression in serous ovarian cancer Microphotographs showing examples of tumors with; **A.** low PDGFβR positive stroma fraction; **B.** high PDGFβR stroma fraction; **C.** low PDGFβR positive perivascular intensity; **D.** high PDGFβR positive perivascular intensity (blue= PDGFβR, red= CD34).

In the absence of the current two-tier grading system a survival sub-analysis was performed on the subgroup (n 165) with moderate and poor differentiation grade (grade 2 and 3), excluding tumors with high differentiation (grade 1). The Kaplan-Meier estimation in patients with grade 2-3 tumor showed a significant reduced survival for the group with high PDGFbR stroma fraction (p=0.022, Log Rank test, median survival 19 months versus 31.3 months, Supplementary Figure 3A). In concordance with the above, high intensity of PDGFβR perivascular staining was found to be correlated with lower overall survival in patients with grade 2-3 tumors, with a median survival of 19.3 months versus 42.3 months as compared to low PDGFβR positive perivascular intensity (p=0.005, Log Rank test, Supplementary Figure 3B). Multivariate analyses confirm the results for lower survival rate both for high PDGFbR positive stroma fraction (HR 1.59, CI 95% 1,07-2,36, p=0.02, Supplementary Table 2) and for high PDGFbR positive perivascular intensity (1.75, CI 95% 1.08-2.82, p=0.02, Supplementary Table 1). Examples of tumor tissues with different PDGFβR status are shown in Figure 3.

The distribution of patients with high and low PDGFβR positive stroma fraction and perivascular intensity did not differ significantly according to the clinico-pathological characteristics of the patients (Supplementary Table 3).

Together these analyses thus demonstrate previously unrecognized associations between high PDGFβR expression in the tumor stroma and survival in serous ovarian cancer.

## DISCUSSION

This study analyzed the stroma of primary tumors and metastasis of serous ovarian cancer using a novel multiparametric approach employing digitalized image analysis. Correlation analysis of stroma features in the primary tumors revealed that α-SMA and PDGFβR-positive cells are largely independently expressed. Notably, perivascular status was neither strongly correlated with vessel density nor size, suggesting that these properties of vascular biology are independently regulated. We also found, in intra-patient comparisons, that most stroma and vessel characteristics differ to a large degree between the primary tumor and the metastatic lesions, except for the status of PDGFβR-positive fibroblasts and perivascular cells that were more concordant. Moreover, high intensity of perivascular PDGFβR staining and abundant PDGFβR-positive stroma were associated with shorter overall survival.

The largely independent expression of α-SMA and PDGFβR in the primary ovarian tumor may indicate that cells expressing these markers constitute functionally distinct subsets. Further studies are warranted to experimentally test this notion. However, it can be noted that recent mouse model studies have suggested that a α-SMA-positive subset of CAFs in pancreas cancer exerts tumor-restraining effects, whereas PDGFβR-positive fibroblast in the present study, as in other reports, has been consistently linked to poor prognosis [28, 29]. The strong correlation between perivascular and fibroblast-like PDGFβR-positive cells found in our study, suggests a shared cell-of-origin of these cell populations. Notably, some lineage-tracing studies in fibrosis and brain scarring models have implied a perivascular cell-of-origin for interstitial fibroblast and for glial cells [30, 31].

Comparisons of stroma characteristics in primary tumors and metastatic sites revealed a large degree of intra-patient variations. Whereas vessel density was not strongly conserved, stromal PDGFβR status in both stromal and perivascular cells displayed a greater degree of stability. This indicates that the PDGFβR related stroma biology is particularly strongly influenced by the genetic make-up of the malignant cells and thus is kept stable regardless of host organ influence. Experimental studies should be done to further explore this hypothesis. While intra-patient genetic differences between primary tumors and metastatic sites are now well established [27, 32], the concordance of stroma features in paired primary tumors and metastasis has been less well studied. However, there are analyses of breast and colorectal cancer that have also suggested that different stroma characteristics vary with regard to their stability in intra-patient comparisons of primary tumors and metastatic lesions [33].

Vascular features, including the status of pericytes, are potentially associated with response to anti-angiogenic drugs [34, 35]. Therefore it is important to consider the large disconcordance in most vascular characteristics in ongoing efforts to identify biomarkers for emerging anti-angiogenic therapies for ovarian cancer such as bevacizumab, pazopanib and nintedanib. Importantly, the present study suggests that vascular features of target metastatic lesions cannot be deduced from analyses of primary tumors but rather need to be analyzed on biopsies from the metastases.

When analyzing the potential impact of the stroma markers on overall survival, we found that a high PDGFβR positive stroma fraction is related to lower survival rate, also after adjusting for clinical prognostic factors. Our finding is in line with reports in recent publications showing a negative impact of PDGFβR positive stroma on survival in breast [36] and prostate cancer [37]. PDGFβR signaling is involved in fibroblast recruitment and activation during developmental and physiological processes [38]. Experimental studies have demonstrated stimulatory effects of PDGF-activated fibroblast both on tumor growth as well as metastasis, and animal models indicate that inhibition of PDGF-signaling in CAF may control tumor growth [39-41]. A series of studies have also shown that PDGFR-signaling in fibroblasts inhibits tumor drug uptake, and thereby negatively regulates therapeutic efficacy [42-44].

Our finding of associations between poor prognosis and high perivascular PDGFβR constitute the first example of a perivascular marker linked to survival in ovarian cancer. Studies of the role of PDGFβR-positive perivascular cells in tumorigenesis have focused largely on vessel maturation [45]. In contrast with our findings concerning ovarian cancer, our group has recently found that low perivascular expression of PDGFβR was associated with shorter survival in metastatic colorectal cancer (Mezheyeuski *et al.*, manuscript) implying that the effect of pericyte expression of PDGFβR may vary in different tumor types.

Our findings on the prognostic impact of protein expression of PDGFβR in the perivascular and stroma fraction of serous ovarian cancer with differentiation grade 2-3 prompted us to make some comparisons with analyses of PDGFRB gene expression in high-grade ovarian cancer in publicly available databases (see Supplementary Files). Analyses of three of the largest databases available revealed variable results regarding associations between high PDGFRB gene expression and overall survival in high-grade serous ovarian cancer (Supplementary Figure 4). One dataset displayed a significant negative impact of high PDGFRB gene expression on survival [46], while one showed a trend for the same result and the third (TCGA) showed no association to survival [47, 48]. The potential explanation for this less strong signal is that the gene expression data rely on material derived from the whole tumor tissue, including all cell types, while our study provides localization data of PDGFβR IHC expression, in stroma fibroblasts and on perivascular cells.

Experimental therapy studies in models of ovarian cancer have explored the effects of dual targeting of endothelial cells and PDGFβR-dependent pericytes. In clinical trials in ovarian cancer, new molecules targeting tumor stroma, including PDGFR, are ongoing. Based on pre-clinical findings linking perivascular status to sensitivity to e.g. VEGF-targeting agents [34, 49], it appears that further exploration of perivascular makers should be carried out to evaluate their potential role as predictive markers for new anti-angiogenic drugs in the treatment of ovarian cancer.

The design of the present study failed to stringently separate the impact of the stromal markers on the natural course of the disease and response to treatment. It is also noted that this somewhat older cohort is characterized by less aggressive surgery than is presently considered as state-of-the-art. At the time of enrolling the study population, the histological grading system was the three-tier system and not the current two-tier system. Nonetheless, as described in the results, we performed an analysis of the subgroup of grade 2 and 3 patients that represent the largest part of the whole cohort. According to the literature, the vast majority of grade 2 and 3 are found in the high-grade category in the two-tier grading system of serous ovarian cancer [4]. It is worth noting that the favorable methodological aspects of the present study include the long follow-up, the use of digital-image-analyses-supported scoring and the analysis of more than one tumor core per case.

In summary, the study identified a previously unrecognized expression pattern of perivascular cells and fibroblasts and revealed that the PDGFβR expression pattern is fairly well conserved in primary tumors and metastases in contrast to the other stroma markers. Moreover, analyses identified PDGFβR expression in perivascular cells and in fibroblasts as possible novel prognostic markers. Our findings suggest that PDGFβR could be explored as a target for personalized tumor microenvironment as especially pericyte targeted treatments.

## MATERIALS AND METHODS

### Patients

Women diagnosed with ovarian cancer 1986 to 2006 were consecutively enrolled at the Department of Gynecologic Oncology, University Medical Center Groningen (Groningen, The Netherlands). Tumor specimens from 355 patients were collected. Of the 355 patients, 186 patients (52%) fulfilled eligibility criteria and were included in the study. The inclusion criteria were: chemo-naive ovarian cancer specimens obtained at primary surgery and serous histologic subtype (Supplementary Figure 1). Clinico-pathological data were retrieved from medical records. Staging was performed according to FIGO (International Federation of Gynecology and Obstetrics [50]). Classification and grading were performed according to World Health Organization standards [51]. As first line chemotherapy, 84.4% received platinum-based treatment, 8.1% did not receive any chemotherapy, 5.4 % received other than platinum-based chemotherapy and treatment data were missing in 2.2 % of the patient.

All patients gave informed consent. Studies were conducted in accordance with the Declaration of Helsinki principles and Institutional review board policies at University Medical Center Groningen.

### The tissue microarray, TMA

TMAs were constructed as described previously [52]. Paraffin-embedded tissue blocks containing tumor in ovarian, omental and peripheral metastasis tissue and corresponding hematoxylin and eosin (H&E)-stained slides were retrieved from the pathology archives. Tumor specimens were obtained from the primary ovarian site in 138 patients, and matched tissue from metastatic lesion were also obtained from 91 patients (Figure 1). In 48 of 186 patients tumor tissue was obtained from only the metastatic site. TMA cores were selected as representative tumor areas by a pathologist both in the primary site (when available) and in the metastatic tissue. The chosen areas of the tumor were marked on the H&E slides. Next, using these H&E slides for reference, four 0.6 mm2 core biopsies were taken from each tumor specimen and arrayed on a recipient paraffin block using a tissue microarrayer (Beecher Instruments, Silver Spring, MD). One to three tissue blocks per patient were available, taken from different tumor areas (primary site, omentum, peripheral metastasis). Using a microtome, 4-mm sections were cut from each TMA block and applied to aminopropyltriethoxysilane-coated slides. All arrayed samples were H&E stained to confirm the presence of tumor tissue [52].

### Detection of stroma markers by immunohistochemistry

TMA sections were deparaffinized in xylene and rehydrated through graded concentrations of ethanol to distilled water. Sections were boiled in a decloaking chamber (Biocare Medical), 110°C for 5 minutes, in pH= 10.0 buffer for PDGFβR and pH=9 buffer for alpha smooth muscle actin and desmin (Dako Target Retrieval Solution) to allow antigen retrieval, and thereafter allowed to cool for 30 minutes. Antigen was blocked with blocking solution (Protein Block Serum-Free Ready-To-Use Dako) for 25 minutes in a humidity chamber at room temperature. Sections were incubated with primary antibody over night at 4°C in humidity chamber. Primary antibodies used were recognizing α-SMA (anti human Smooth Muscle Actin, code M0851, Clone 1A4; Dako, Inc., Denmark (dilution 1:300)), PDGFβR (PDGF Receptor beta 28E1 Rabbit mAb, 3169, Cell Signaling Technology, Danvers, MA (dilution 1:70)) and Desmin (Rabbit Anti-Human Desmin code HPA 018803-100UL Sigma Life Sciences, St Louis, MO (dilution 1:500)).

Sections were then incubated with secondary anti-mouse or anti-rabbit antibody (ImmPRESS™-AP Polymer Anti-Mouse IgG, MP-5402 and ImmPRESS™-AP Polymer Anti-Rabbit IgG MP-5401, Vector Laboratories, Burlingame, CA) for one hour at room temperature. Section were washed for 5 minutes twice in PBS-T (Phosphate buffered saline - 0.1% Tween 80), and once in Tris acetate buffer 0.2 M Tris acetate 0.005M EDTA pH 8.1 for 5 minutes, and developed with Vector Blue AP substrate Kit (SK-5300, Vector Laboratories, Burlingame, CA) using the same Tris acetate buffer with 0.07 g of NaCl per 5 ml of the solution.

Sections were then again denatured in decloaking chamber at 90°C for 5 minutes, with pH=9 solution, blocked in blocking solution for 25 minutes in humidity chamber and incubated with primary antibody against CD34 (Clone JC70A; Dako, Inc., Denmark (dilution 1:100)) for 1 hour at room temperature. Sections were then incubated with ImmPRESS-AP Alkaline Phosphatase Polymer Anti-Mouse Kit at room temperature in a humidity chamber. Following washes in PBS-T for 5 minutes twice and once in Tris acetate buffer (described above) for 5 minutes, and developed with Vector Red AP substrate Kit (SK-5100, Vector Laboratories, Burlingame, CA) using the Tris acetate buffer with NaCl described above. Sections were finally mounted with aqueous mounting media.

### Digital image analyses

The double stained slides were scanned and, after quality selection, images were analyzed using Image J software, with an algorithm developed in-house (see Supplemental Material and Methods for details). CD34 staining was used to determine vessel density, mean vessel area and mean vessel perimeter. For perivascular-restricted measurements the areas surrounding the vasculature were analyzed. Analyses of desmin-, PDGFβR- and α-SMA-stained samples yielded information about average intensity of the staining with these three markers in the perivascular area (perivascular intensity). Perivascular intensities of individual vessels were measured in optical density values (OD), (for details see Supplementary Files). To obtain values for perivascular fraction, individual vessels were classified as ‘uncovered’ (OD value below or equal to the 10% of maximal detected intensity) or ‘covered’ (OD value above 10% of maximal detected intensity) and ratio of covered vessels over total vessels per case was thereafter calculated, to yield the perivascular fraction metric.

PDGFβR- and α-SMA-staining were also used to determine the stroma fraction: the fraction of total tumor area positive for these markers. The marker-positive area was defined as the sum of regions which had a pixel intensity above a pre-set background value. Small regions (up to 15 square micrometers, or 50 square pixels) with pixel intensity above the threshold and all regions below the threshold were categorized as marker-negative. The threshold was set after evaluation of a set of 10 randomly selected images. If the small ‘negative’ regions (with minimal linear dimension up to 11 micrometers, or 20 square pixels) appear inside ‘positive’ areas, the former were considered as positive. The stroma fraction was calculated as the sum of all positive regions divided by the total tumor area. In the case of PDGFβR analyses this step also included exclusion of 35 cores with positive epithelial staining. Finally, PDGFβR and α-SMA-staining were used to obtain values for PDGFβR- and α-SMA - intensity (stroma intensity) by calculating the average intensity of PDGFβR- and α-SMA-staining in the marker-positive area.

Together these analyses, performed on the CD34/α-SMA, CD34/desmin and CD34/PDGFβR-staining yielded quantitative data for 13 different stroma-related metrics (Figure 1).

### Statistical analysis

The Spearman two-tailed test was used for correlation estimation between stromal markers expression, a correlation coefficient of 0.5 and a *p*-value <0.01 were used as reference threshold values. Cox proportional hazards model and the Kaplan-Meier estimator were used to analyze the association between the markers and overall survival (OS). Kaplan Meier survival analysis was used to analyze survival rates and a multivariate Cox regression model was used to calculate hazard ratios of the clinical-pathological factors and the stroma related metrics for patients survival and to determine their independence. The survival findings were confirmed by backward selection. Associations between stroma metrics and clinico-pathological characteristics of the patients were performed with Chi-square test. All tests were done at the 95% significance level and were performed using SPSS version 22 (SPSS Inc., Chicago, IL). Forest Plot was done using R 3.2.2. meta package.

## SUPPLEMENTARY MATERIALS AND METHODS FIGURES AND TABLES


