# Supplementary material for: Markers of fibroblast-rich tumor stroma and perivascular cells in serous ovarian cancer: Inter- and intra-patient heterogeneity and impact on survival
Source: Oncotarget. 2016 Feb 23;7(14):18573–84. doi: 10.18632/oncotarget.7613 (PMC4951310; doi:10.18632/oncotarget.7613)
Supplement: Supplementary file 1 [file oncotarget-07-18573-s001.pdf]

SUPPLEMENTARY MATERIALS AND METHODS

Digital image analyses

Slide scanning was performed with a Vslide slide-scanning microscope (Metasystems, Alltlussheim, Germany) using ×10 objective and RGB led illumination for color deconvolution. The software Metaviewer (Metasystems, Alltlussheim, Germany) was used to view the scanned images, which were then manually annotated and saved in TIFF format as individual images.

The images underwent an automated image analysis platform. An image-processing algorithm has been developed as a plugin for the ImageJ software (http://rsb.info.nih.gov/ij). The algorithm to identify vessels utilized the specific endothelial staining, performed with an antibody against CD34.

Perivascular areas were defined as the 5.5 micrometers thick perimeter surrounding each CD34 positive area. The intensity of the staining of the different perivascular markers, in each perivascular area, was then defined (see below). "Perivascular intensity" for each case was calculated as the mean intensity of all perivascular areas for each case. "Fraction covered vessels" for each case was calculated as the fraction of vessels displaying an intensity of at least 10% of that of the highest value.

The intensity was measured in a scale of 256 grades (from 0 to 255) because of the RGB-based 8-bit format of the images used in the study. The intensity is proportional to the amount of light transmitted through the slide. However, since the stained tissue absorbs the light, the amount of the staining dye in the tissue is equivalent to the absorbance. The Beer–Lambert's law was used to calculate optical density (OD) of each area that corresponds to the amount of light absorber.

Online public gene expression databases

Three of the largest publicly available gene expression databases of ovarian cancer were used. The download of the datasets in R was done through the Bioconductor package [53]. Only data from the subgroup of high-grade serous ovarian cancer patients were used for analyses. Cox regression correlation analyses of PDGFβR gene expression levels and overall survival (OS) were performed in SPSS. The Tothill et al. dataset, GSE989 [46] contains expression profiles for 156 high-grade serous ovarian cancer patient samples; the TCGA dataset, TCGA_eset [48] contains expression profiles from 480 high-grade serous ovarian cancer patient samples and the Yoshihara et al. dataset, GSE32062.GPL6480, contains expression profiles from 129 high-grade serous ovarian cancer patient samples [47].

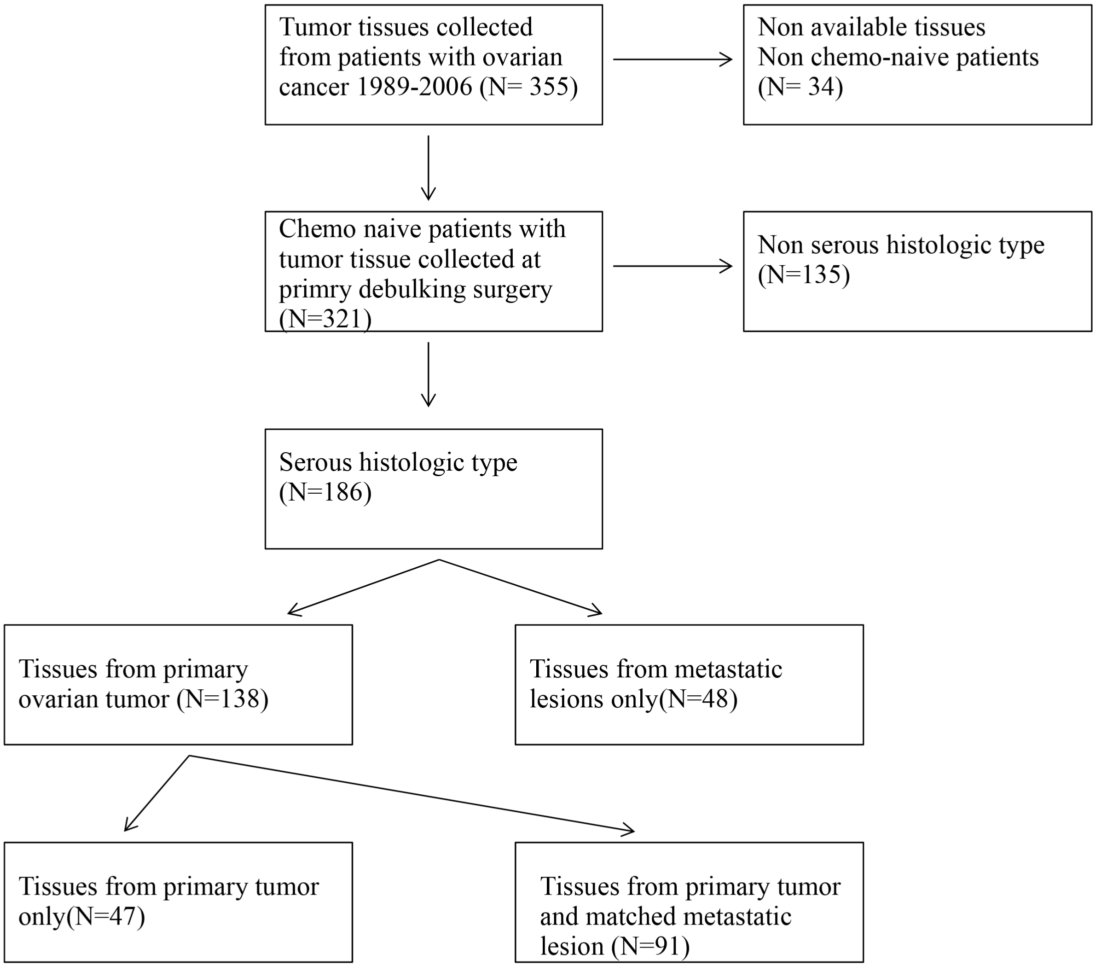

Supplementary Figure S1: Consortium diagram of the study population.

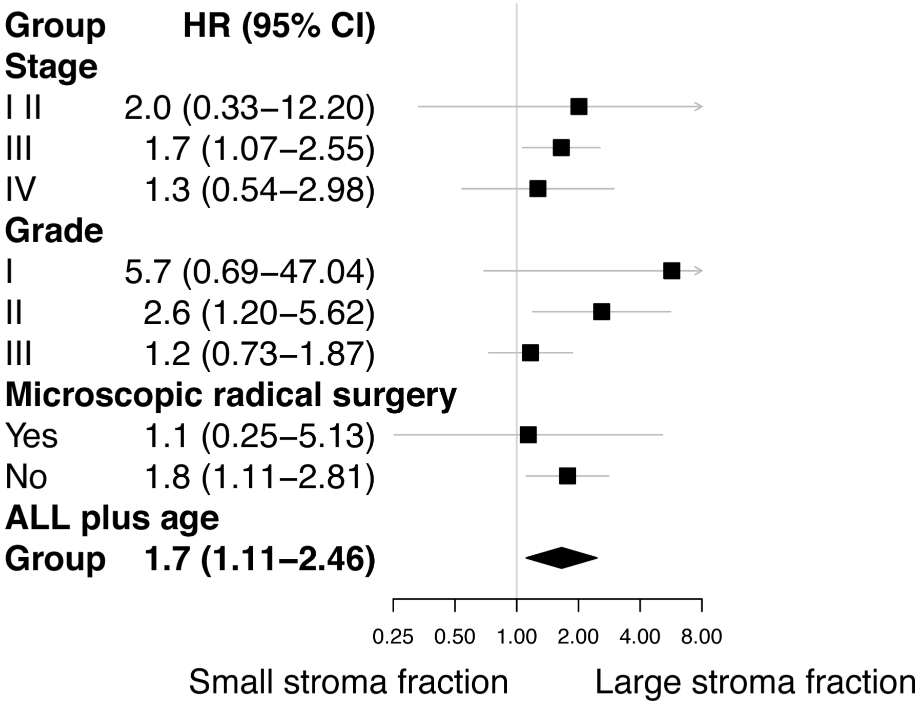

Supplementary Figure S2: Forest Plot of PDGFβR positive stroma fraction in clinico-pathological subgroups. The relative impact on overall survival, displayed as hazard ratio (HR) of the PDGFβR positive stroma fraction differs according to the clinico-pathological subgroups.

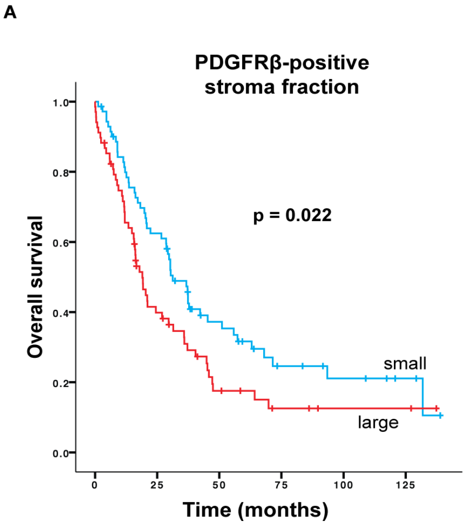

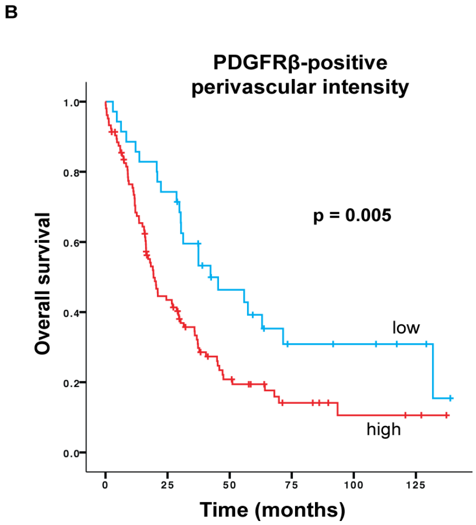

Supplementary Figure 3: Survival curves for high and low PDGFβR in the subgroup of serous ovarian cancer patients with grade 2 and 3. A. Kaplan-Meier graph shows worse overall survival for high PDGFβR positive stroma fraction as compared to low PDGFβR positive stroma fraction in serous ovarian cancer with grade 2-3 (n=165 patients) (p=0.022, Log Rank). Median survival for high PDGFβR positive stroma fraction is 19.1 months, versus 31.3 months for low PDGFβR positive stroma fraction. **B.** Survival curves for high and low PDGFβR positive perivascular intensity in the subgroup of serous ovarian cancer patients grade 2-3. Kaplan-Meier graph shows worse overall survival for high PDGFβR positive perivascular intensity as compared to low PDGFβR positive perivascular intensity, in 165 patients (p=0.005, Log Rank). Median survival for high PDGFβR positive perivascular intensity was 19.3 months versus 42.3 months for low PDGFβR positive perivascular intensity.

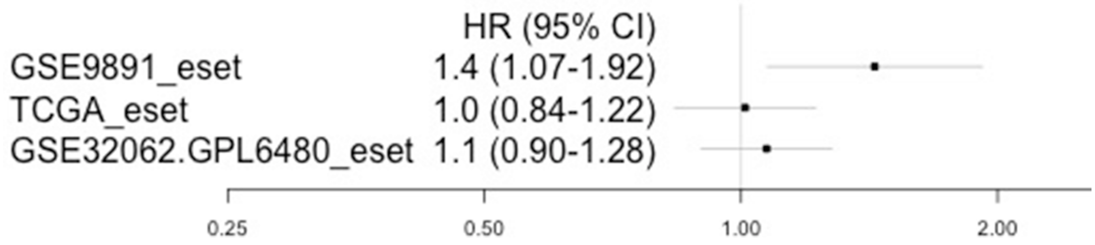

Supplementary Figure 4: Relationships between PDGFRB gene expression and overall survival in high-grade serous (HGS) ovarian cancer. Impact on overall survival, displayed as hazard ratio (HR), of PDGFRB gene expression in HGS ovarian cancers of three cohorts of ovarian cancer with publicly available gene-expression data sets.

Supplementary Table 1: Uni- and multivariate analyses of the impact of each clinical prognostic variable and PDGFβR perivascular intensity on overall survival, excluding grade 1 patients

Variables	Univariate analysis		Multivariate analysis	
	**HR (95% CI)**	***p*-value**	**HR (95% CI)**	***p*-value**
Age at diagnosis	1.01 (0.99-1.03)	0.42	0.99 (0.97-1.01)	0.46
FIGO stage				
I+II	1 (reference)		1 (reference)	
III	5.93 (2.16-16.28)	0.001	2.47 (0.7-8.68)	0.16
IV	9.71 (3.33-28.31)	<0.001	3.98 (1.04-15.3)	0.04
Residual tumor after primary surgery				
No residual tumor	1 (reference)		1 (reference)	
Residual tumor	8.19 (3.26-20.61)	<0.001	3.35 (1.23-9.09)	0.02
PDGFβR positive perivascular intensity				
Low PDGFβR	1 (reference)		1 (reference)	
High PDGFβR	1.95 (1.22-3.11)	0.005	1.75 (1.08-2.82)	0.02

Abbreviations: HR=hazard ratio, CI=confidence interval

Supplementary Table 2: Uni- and multivariate analyses of the impact of each clinical prognostic variable and PDGFβR positive stroma fraction on overall survival, excluding grade 1 patients

Variables	Univariate analysis		Multivariate analysis	
	**HR (95% CI)**	***p*-value**	**HR (95% CI)**	***p*-value**
Age at diagnosis	1.01 (0.99-1.03)	0.42	0.99 (0.97-1.01)	0.32
FIGO stage				
I+II	1 (reference)		1 (reference)	
III	5.93 (2.16-16.28)	0.001	2.29 (0.64-8.23)	0.20
IV	9.71 (3.33-28.31)	<0.001	3.65 (0.93-14.35)	0.06
Residual tumor after primary surgery				
No residual tumor	1 (reference)		1 (reference)	
Residual tumor	8.19 (3.26-20.61)	<0.001	3.82 (1.37-10.68)	0.01
PDGFβR positive stroma fraction				
Low PDGFβR	1 (reference)		1 (reference)	
High PDGFβR	1.57 (1.06-2.32)	0.02	1.59 (1.07-2.36)	0.02

Abbreviations: HR=hazard ratio, CI=confidence interval

Supplementary Table 3: Clinico-pathological characteristics of patients and their association with PDGFβR positive stroma fraction and perivascular intensity (Chi square, Asymptotic test)

Clinical variable	Cases n (%) n=186	PDGFβR stroma fraction		PDGFβR perivascular intensity	
FIGO stage		**LOW**	**HIGH**	**LOW**	**HIGH**
I	10 (5.4%)	4 (66.7%)	2 (33.3%)	2 (33.3%)	4 (66.7%)
II	13 (7.0%)	4 (40%)	6 (60%)	3 (30%)	7 (60%)
III	130 (69.9%)	55 (50%)	55 (50%)	28 (25.5%)	82 (74.5%)
IV	32 (17.2%)	12 (46.2%)	14 (53.8%)	4 (15.4%)	22 (84.6%)
p value		0.75		0.65	
Diff. grade					
1 (high)	21 (11.3%)	5 (35.7%)	9 (64.3%)	3 (21.4%)	11 (78.6%)
2 (mod.)	51 (27.4%)	21 (53.8%)	18 (46.2%)	12 (30.8%)	27 (69.2 %)
3 (low)	98 (52.7%)	46 (51.1%)	44 (48.9%)	20 (22.2%)	70 (77.8%)
p.value		0.61		0.73	
Radical surgery					
YES	27 (14.5%)	11 (52.4%)	10 (47.6%)	7 (33.3%)	14 (66.7%)
NO	102 (58.4%)	43 (46.7%)	49 (53.3%)	18 (19.6%)	74 (80.4%)
p-value		0.66		0.18	

Abbreviation: Diff. grade: differentiation grade
